# High Internal Phase Emulsions Stabilized with Ultrasound-Modified Spirulina Protein for Curcumin Delivery

**DOI:** 10.3390/foods13091324

**Published:** 2024-04-26

**Authors:** Qing Liu, Tao Chen, Lihang Chen, Runan Zhao, Ximei Ye, Xinchuang Wang, Di Wu, Jiangning Hu

**Affiliations:** 1 State Key Laboratory of Marine Food Processing and Safety Control, Dalian Polytechnic University, Dalian 116034, China; 15670045469@163.com (Q.L.); 13084128062@163.com (T.C.); lhchen@xy.dlpu.edu.cn (L.C.); 18851905610@163.com (X.Y.); wxc_0627@163.com (X.W.); wudisp@dlpu.edu.cn (D.W.); 2 School of Food Science and Technology, Dalian Polytechnic University, Dalian 116034, China; 3 College of Biosystems Engineering and Food Science, Zhejiang University, Hangzhou 310058, China; zrn951219@163.com

**Keywords:** high internal phase emulsion, spirulina protein, protein structure, ultrasound, curcumin, bioavailability

## Abstract

Spirulina protein (SP) is recognized as a nutritious edible microbial protein and holds potential as a natural emulsifier. Due to the inherent challenges SP faces in stabilizing high internal phase emulsions (HIPEs), ultrasonic techniques were utilized for modification. Noticeable alterations in the structural and functional properties of SP were observed following ultrasonic treatment at various power levels (0, 100, 300, and 500 W). Ultrasound treatment disrupted non-covalent interactions within the protein polymer structure, leading to the unfolding of molecular structures and the exposure of hydrophobic groups. Importantly, the particle size of SP was reduced the most at an ultrasonic power of 300 W, and the three-phase contact angle reached its peak at 84.3°. The HIPEs stabilized by SP modified with 300 W ultrasonication have high apparent viscosity and modulus values and strong storage stability under different environmental conditions. Additionally, the encapsulation of curcumin in HIPEs led to improved retention of curcumin across various settings. The bioavailability increased to 35.36, which is 2.8 times higher than the pure oil. These findings suggest that ultrasound-modified SP is a promising emulsifier for HIPEs, and is expected to encapsulate hydrophobic nutrients such as curcumin more effectively.

## 1. Introduction

Curcumin is a polyphenolic compound extracted from Curcuma longa rhizomes [1] that exhibits multiple functional properties, including antioxidant, anticancer, and hypolipidemic effects [2,3,4]. However, curcumin’s poor water solubility and low chemical stability significantly reduce its bioavailability [5], limiting its pharmaceutical and food industry applications. To address this, several stabilization systems have been developed, with emulsions being a cost-effective solution [6]. Given the hydrophobic nature of curcumin, oil-in-water emulsions are particularly beneficial for its delivery.

High internal phase emulsions (HIPEs) are characterized by an internal phase volume fraction exceeding 0.74, making them super-concentrated emulsions. This concentration leads to tightly packed emulsion droplets that form a three-dimensional network, imparting solid-like properties to the HIPEs [7]. Traditionally, HIPEs have relied on high concentrations of surfactants for stabilization [8], which can be costly, environmentally detrimental, and pose food safety issues, rendering them unsuitable for food applications. Consequently, the search for green, safe, and economical natural emulsifiers as alternatives to conventional surfactants has become imperative. Recent studies have highlighted starch, polysaccharides, and proteins as promising natural emulsifiers for HIPEs [9]. Among these, protein-stabilized HIPEs have gained considerable attention due to their safety, stability, and potential applications in the food sector [10]. However, most of the current studies have focused on animal and plant proteins [11,12,13], whereas microbial protein-stabilized HIPEs remain underexplored.

Spirulina platensis, an edible, spiral, multicellular cyanobacterium that possesses antioxidant, anti-inflammatory, and antimicrobial properties, has been widely used in food, pharmaceutical, and cosmetic applications [14,15]. Spirulina, with its high protein content (65–70%) and essential amino acids [16], offers significant health benefits. However, fewer studies have been conducted on the emulsification properties of spirulina proteins (SPs). In previous research, we observed that SP-based HIPEs exhibited poor stability. Therefore, it is necessary to enhance the emulsification properties of SP to improve its application value. Protein modification can be achieved through physical, chemical, and enzymatic methods [17]. Among these, ultrasound technology, a non-thermal, safe, and non-toxic physical method, can alter proteins’ structural and functional properties due to its unique cavitation effect, which generates shear forces, turbulence, and shock waves [18,19]. Prior research by Ma et al. [20] reported increased hydrophobicity and improved emulsion aggregation in ultrasonically treated cod protein. Similarly, Zuo et al. [21] documented enhanced stability in HIPEs using ultrasound-treated quinoa proteins.

**Scheme 1:**
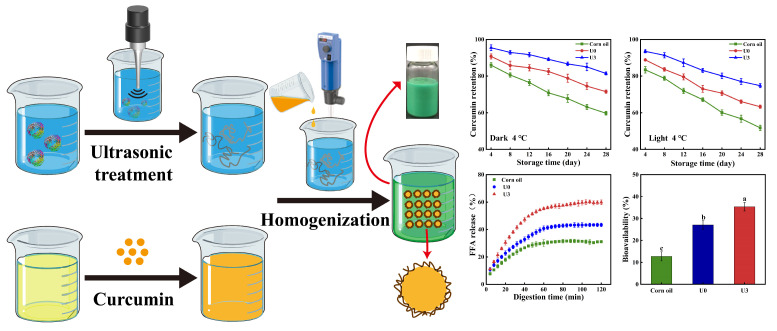
Schematic representation of the effect of ultrasonication of SP on the preparation of HIPEs for curcumin encapsulation.

The aim of this study was to treat SP with ultrasound to improve its properties necessary for the development of stable HIPEs with curcumin as an active compound. The impacts of various ultrasound power settings on the structural and functional characteristics of SP were explored. Subsequently, HIPEs were formulated using ultrasonicated SP as an emulsifier with corn oil, and their microstructure, rheological characteristics, and stability were examined. Additionally, the study assessed the storage stability of HIPEs under various environmental conditions and their influence on the protective and bioavailability properties of curcumin (Scheme 1). The findings of this study may provide new insights into the development of natural emulsifiers and encapsulated delivery systems for hydrophobic natural nutrients.

## 2. Materials and Methods

### 2.1. Materials

Spirulina powder was purchased from Tian Jian Biological Co., Ltd. (Binzhou, China). Corn oil was purchased from a local supermarket in Dalian, China. Curcumin, 5, 5′-dithio-bisnitrobenzoic acid (DTNB), 8-anilino-1-naphthalenesulfonic acid (ANS), pepsin, and lipase were procured from Macklin Biological Co., Ltd. (Shanghai, China). The BCA protein assay kit, ethylenediaminetetraacetic acid (EDTA), and trypsin were procured from Beijing Solarbio Science & Technology Co., Ltd. (Beijing, China). Bile salt was obtained from Qingdao Hope Biotechnology Co., Ltd. (Qingdao, China). Other reagents were analytical grade.

### 2.2. Ultrasonic Treatment of SP

All SP used in this paper were extracted by our laboratory. Protein dispersion was prepared by dissolving 1 g of SP in 100 mL of deionized water. The pH of the protein dispersion was adjusted to 7.0 using 1 M HCl and NaOH, and the solution was stirred continuously for two hours. A total of 70 mL of this SP dispersion was then placed in an ice bath and subjected to ultrasonic treatment using an ultrasonic cell crusher (JY92, Ningbo, China) equipped with a 6 mm probe, operating at a frequency of 20 kHz. The ultrasonic power was set to 0, 100, 300, and 500 W, and the ultrasonic time was set to 15 min (working for 2 s, stopping for 2 s). The SPs treated with different ultrasonic powers were named U0, U1, U3, and U5, respectively.

### 2.3. Characterization of SP

#### 2.3.1. Three-Phase Contact Angle (θ)

The three-phase contact angle (θ) of SP was measured with a droplet-shaped analyzer (DSA25, KRÜSS, Hamburg, Germany). The SP particles were pressed into 2 mm thick tablets and soaked in corn oil for 10 min. Subsequently, the tablets were removed, and deionized water was slowly added to their surfaces. The shape of the resulting droplets was then recorded using a high-speed camera [22].

#### 2.3.2. Transmission Electron Microscopy (TEM)

The ultrastructure of SP was observed using TEM (JEM-2100, JEOL, Tokyo, Japan). In this experiment, a certain concentration of SP solution was carefully added to a carbon film with a copper mesh. The sample was then left to dry naturally at room temperature. Once dried, the sample was observed using TEM. The average particle size of SP was statistically analyzed by counting the sizes of >100 individual particles in their TEM images using ImageJ 1.53K software (National Institutes of Health, Bethesda, MD, USA).

#### 2.3.3. Surface Hydrophobicity

To assess the surface hydrophobicity of the SP, various concentrations (ranging from 0.01 to 0.1 mg/mL) were prepared. These solutions were then combined with a phosphate buffer (pH 7.0, 10 mM) containing 8 mM ANS. The resulting mixture was incubated in the dark for 15 min. The fluorescence intensity of the samples was measured using a fluorescence spectrophotometer (Hitachi F-2700, Tokyo, Japan), with emission and excitation wavelengths set at 470 nm and 390 nm, respectively. Surface hydrophobicity was calculated from the initial slope of the relative fluorescence intensity and protein concentration [23].

#### 2.3.4. Free Sulfhydryl Group

Free sulfhydryl content was determined according to the method previously described and performed by Wang et al. [24] with slight modifications. An aliquot of each sample was diluted to a protein concentration of 0.1% using Tris-Gly buffer (0.086 M Tris-HCl, 0.09 M Gly, and 4 mM EDTA, pH 8.0). Then, 0.08 mL of DTNB solution at a concentration of 4 mg/mL was added and vortexed to mix (except for the blank). The mixed solution was immediately incubated at room temperature in the dark for 30 min, and the absorbance of the supernatant was measured at 412 nm using a Lambda 35 ultraviolet spectrophotometer (PerkinElmer, Cambridge, MA, USA). The free sulfhydryl content of the SP solution was calculated according to the following Equation (1):

Free sulfhydryl group (μmol/g) = (73.53 × A_412_ × D)/C (1)

where A_412_ is the absorbance of the sample at 412 nm, D is the dilution factor, and C is the SP solution concentration (mg/mL).

#### 2.3.5. Protein Solubility

The solubility of SP was determined by the method described by Chen et al. [25] with appropriate modifications. A BCA total protein quantification kit was used to determine the protein concentration. The SP solution was centrifuged at 10,000 r/min for 15 min to obtain the supernatant. Protein solubility (%) is the ratio of protein concentration in the solution before and after centrifugation.

#### 2.3.6. Ultraviolet–Visible (UV–Vis) Spectrum

The UV–Vis spectrum of SP was measured at 250–800 nm using a UV spectrometer (Hitachi-UH5300, Tokyo, Japan).

#### 2.3.7. Endogenous Fluorescence Spectrum

The fluorescence spectrum of SP was determined using a fluorescence spectrophotometer (Hitachi F-2700, Tokyo, Japan). The excitation wavelength was set to 280 nm and the emission wavelength was set to 300–500 nm. The excitation and emission slit widths were set to 5 nm.

#### 2.3.8. Circular Dichroism (CD) Spectrum

The CD spectrum of SP in the wavelength range of 200–240 nm was measured using a CD spectrometer (J-1500, JASCO, Oklahoma City, OK, USA), which was used to analyze the secondary structure changes of SP.

### 2.4. Preparation of HIPEs

Oil-in-water HIPEs with an oil-phase volume fraction (φ) of 0.75 were prepared using a homogenizer (T25, IKA, Staufen, Germany). SP-stabilized HIPEs were prepared by mixing the SP dispersion with corn oil and homogenizing at a steady speed of 15,000 r/min for 1.5 min.

### 2.5. Characterization of HIPEs

#### 2.5.1. Confocal Laser Scanning Microscopy (CLSM)

CLSM measurements were conducted according to the method described by Li et al. [26]. A mixed fluorescent dye solution of 1 mg/mL was prepared using equal amounts of Nile Red and Nile Blue. Subsequently, 1 mL of this dye solution was used to stain the sample for 10 min. A drop of the stained emulsion was then dispersed onto a laser confocal sample box and spread evenly. The microstructure was observed using a confocal laser scanning microscope (SP 8, LEICA, Wetzlar, Germany), with the argon-krypton laser excitation wavelength set at 488 nm and the helium-neon (He/Ne) laser excitation wavelength at 633 nm.

#### 2.5.2. Morphological Measurements of HIPEs

Oil droplets in HIPEs were observed using an optical microscope (DM2500, LEICA, Wetzlar, Germany). Drops of the emulsion were placed on slides and observed under a 10× objective lens to analyze the morphology of the emulsion samples and capture images using a camera. The size of the oil droplets in the microscopic images was calculated using ImageJ software to determine the particle size distribution of the emulsion samples.

#### 2.5.3. Centrifugation Stability

A total of 5 mL of HIPEs was added to a 10 mL centrifuge tube. The samples were centrifuged for 10 min (4 °C, 10,000 r/min) using a high-speed centrifuge (CF16RXII, Hitachi). After centrifugation, the appearance of the HIPEs was recorded photographically.

#### 2.5.4. pH and Ionic Strength Stability

The effect of pH on the emulsion was explored by adding 1 M of HCl and NaOH solution to the protein solution and adjusting the final pH of the emulsion (2~9). The ionic strength of sodium chloride was used at 0, 200, 400, 600, and 800 mM. The emulsions were stored at 4 °C and the appearance and micromorphology of the emulsions were recorded at 0 days and 14 days.

#### 2.5.5. Rheological Analysis

The rheological properties of HIPEs were determined using the method of Zuo et al. [27]. At a temperature of 25 °C, the rheometer equipped with a flat aluminum fixture measuring 40 mm in diameter was employed to analyze the rheological characteristics of HIPEs (Discovery HR-2, TA, New Castle, DE, USA). The storage modulus (G′) and loss modulus (G″) of the emulsions were scanned over a range of oscillation frequencies from 0.1 to 10 Hz. The apparent viscosity of the emulsions was determined over a range of shear rates from 0.1~100 s^−1^.

### 2.6. Preparation of Curcumin-Loaded HIPEs

A total of 0.03 g of curcumin was added to 100 mL of corn oil and stirred overnight. HIPEs encapsulating curcumin were prepared by combining curcumin dissolved in corn oil with SP dispersion according to 2.4. The concentration of curcumin in the HIPEs was determined from a curcumin standard curve: 0.2 mL of curcumin-encapsulated HIPEs were vortexed with 4.8 mL of di-chloromethane and centrifuged at 10,000 r/min for 10 min. The absorbance of the supernatant was measured at 420 nm using a Lambda 35 ultraviolet spectrophotometer (PerkinElmer, Cambridge, MA, USA). The curcumin concentration was calculated by substituting the absorbance into the standard curve equation (y = 0.137x + 0.0098, R^2^ = 0.9991).

### 2.7. Retention of Curcumin

Different samples containing curcumin were kept at 4 °C in dark and light for 28 days. The curcumin content was determined every 4 days. Curcumin retention was calculated as follows [28]:

Curcumin retention (%) = Ct/C_0_ × 100% (2)

where Ct is the curcumin concentration in the emulsion after storage for a certain period, t is the corresponding number of days, and C_0_ is the curcumin concentration in the fresh emulsion.

### 2.8. In Vitro Simulated Digestion of HIPEs

The samples were mixed with artificial saliva at a ratio of 1:1 for 10 min at 37 °C and then mixed with gastric fluid (SGF) and intestinal fluid (SIF) for 2 h at 37 °C, respectively. The gastric and intestinal fluids were prepared as described by Li et al. [26]. After digestion, images of the digested droplets in the emulsion were recorded using a biomicroscope.

#### 2.8.1. Free Fatty Acid (FFA) Release

The pH of the intestinal digestive system was consistently maintained at 7.0 by the dropwise addition of 0.1 M NaOH. The rate of FFA release during intestinal digestion was determined by recording the amount of NaOH consumed. The percentage of FFA release was calculated as follows [28]:

FFA (%) = (V_NaOH_ × C_NaOH_ × M_oil_)/(2 × m_oil_) × 100% (3)

where V_NaOH_ (mL) was the volume of NaOH consumed during the measurement, C_NaOH_ (M) was the molar concentration of NaOH, M_oil_ (g/mol) was the molecular weight of corn oil, and m_oil_ (g) was the total weight of corn oil used in the experiment.

#### 2.8.2. Bioavailability of Curcumin

After the digestion process, the enteric digest was centrifuged at 10,000 r/min for 30 min. Subsequently, the intermediate micellar layer was carefully collected. The concentration of curcumin in both the micellar layer and the enteric digest was determined. The bioavailability of curcumin was then calculated using the following equation [29]:

Bioavailability (%) = (C_Micelles_/C_Initial_) × 100% (4)

where C_Micelles_ is the concentration of curcumin in the micellar layer and C_Initial_ is the concentration of curcumin based on the initial addition.

### 2.9. Statistical Analysis

All graphs were generated by employing Origin 2018 software. All measurements in this study were conducted three times to ensure accuracy and reliability. The results were subjected to analysis of variance (ANOVA) using statistical software (IBM SPSS Statistics 22). Treatment means that exhibited statistically significant differences (*p* < 0.05) were subjected to comparison using the Duncan multiple range (DMR) test.

## 3. Results

### 3.1. Characteristics of SP

#### 3.1.1. The Morphology of SP and the Three-Phase Contact Angle

**Figure 1:**
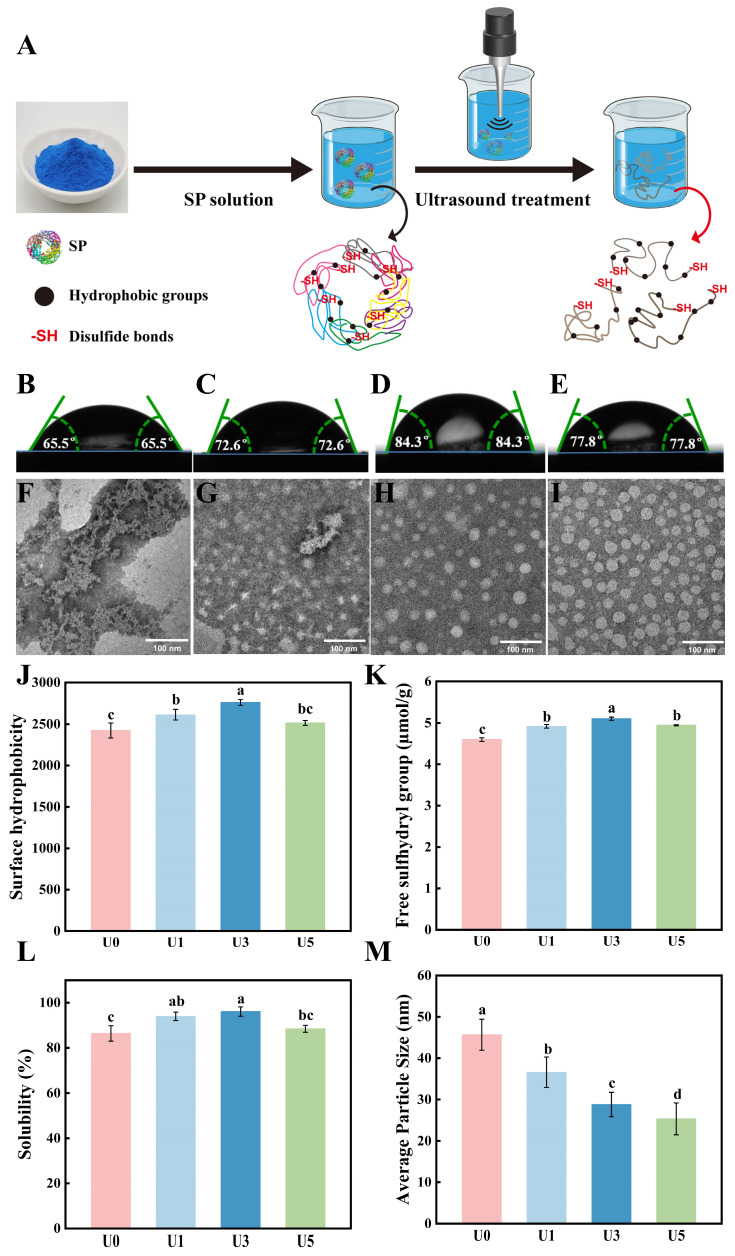
Schematic representation of the effect of ultrasonic treatment to improve the hydrophobicity of SP surfaces (**A**); Three-phase contact angle (**B**–**E**); TEM (**F**–**I**); Surface hydrophobicity (**J**); Free sulfhydryl group (**K**); Solubility (**L**); Average Particle Size (**M**) of SP with different ultrasonic power treatments. From left to right, U0, U1, U3, U5. Different lowercase letters in the column diagram suggest significant differences (*p* < 0.05).

The micromorphological changes in SP treated with different ultrasonic powers are illustrated in Figure 1F–I. It is noteworthy that the shape and size of SP particles changed significantly after treatment. As shown, U0 mainly existed in the form of aggregates. The SP aggregates were gradually dispersed and the particle size decreased after ultrasonication (Figure 1M). These observations suggest that ultrasound disrupts non-covalent interactions within protein aggregates, leading to particle size reduction and morphological changes [27]. Moreover, the wetting ability of SP was evaluated by determining its three-phase contact angle (θ), a vital parameter that impacts the type and stability of emulsions. A θ value closer to 90° indicates that the protein has a stronger adsorption capacity at the oil–water interface at this point and can better stabilize the emulsion [29]. Figure 1B shows that the three-phase contact angle of U0 is 65.5°, which indicates its poor hydrophobicity. The contact angles of the SP treated with various ultrasonic powers all increased to different extents. Among them, the contact angle of U3 was the highest at 84.3°. These findings suggest that the proper ultrasonic treatment not only hinders the aggregation of SP but also exposes more hydrophobic groups within SP.

#### 3.1.2. Surface Hydrophobicity

The surface hydrophobicity of proteins is closely related to their structural stability and emulsification properties. Figure 1J shows the changes in the surface hydrophobicity of SP after treatment with different ultrasound powers. Ultrasonic waves acted on SP to unfold its structure and reduce the particle size (Figure 1M), leading to the exposure of more hydrophobic groups on the surface. This increased surface hydrophobicity, with a peak value of 2759.27 ± 34.74 observed at 300 W. On the other hand, surface hydrophobicity was reduced at an ultrasonic power of 500 W, which was in agreement with the results of fluorescence spectroscopy. Liu et al. [23] reported similar results, which may be due to the partial denaturation or reaggregation of proteins by high-intensity sonication, resulting in the re-burial of hydrophobic groups.

#### 3.1.3. Free Sulfhydryl Groups

Free sulfhydryl groups, as the key functional groups of protein molecules, have a crucial role in the emulsification properties of SP. As shown in Figure 1K, the free sulfhydryl content of SP increased from 4.60 ± 0.05 μmol/g to 5.10 ± 0.04 μmol/g when the ultrasonic power was increased from 0 W to 300 W before decreasing. This may be because high-intensity sonication caused protein depolymerization, resulting in the burial of a portion of free sulfhydryl groups. In addition, a large number of free radicals are generated during high-intensity sonication, which oxidizes the exposed free sulfhydryl groups and decreases their content [24].

#### 3.1.4. Solubility

Solubility is an important indicator for evaluating the functional properties of proteins. As shown in Figure 1L, the solubility of sonicated SP increased compared to non-sonicated samples. This may be because sonication disrupts the noncovalent interactions between proteins, leading to the depolymerization of protein aggregates into smaller particles and increasing the interaction between proteins and water molecules [30]. On the contrary, too much ultrasound power can cause SP molecules to re-aggregate, leading to the decreased solubility of U5. This is consistent with the results of morphological changes in protein.

#### 3.1.5. UV–Vis Spectrum

**Figure 2:**
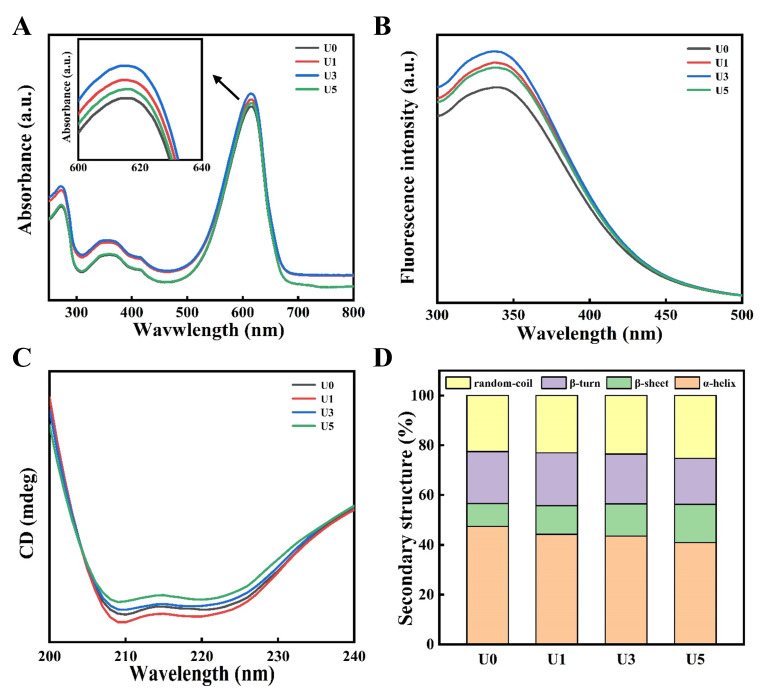
UV–Vis absorption spectra (**A**); Fluorescence spectra (**B**); CD spectra (**C**); The content of the secondary structure (**D**) of SP with different ultrasonic power treatments.

UV–Vis absorption spectroscopy is a valuable tool for assessing alterations in the spatial structure of proteins. External environmental factors can affect a protein molecule, leading to changes in the absorbance intensity of its aromatic amino acid residues (Trp, Tyr, and Phe) in the near-UV region [31]. As shown in Figure 2A, the intensity of the absorption peak at 280 nm increased after sonication. This suggests that ultrasonication may unfold the protein structure and facilitate the exposure of the chromogenic groups [25]. The characteristic absorption peak of SP, located near 620 nm, correlates with the degree of pigment retention within the molecule [32]. The observed increase in peak intensity in this region post-sonication may be indicative of structural changes within the protein.

#### 3.1.6. Endogenous Fluorescence Spectrum

To further investigate changes in the tertiary structure of SP, its endogenous fluorescence spectra were analyzed [33]. Figure 2B illustrates that sonication enhanced the fluorescence intensity of protein molecules to varying degrees. This enhancement suggests that ultrasonication can expose more chromophores (Trp chromophores) within SP, leading to varying degrees of conformational changes in the protein [34]. Similar findings were reported by Kong et al. [35] in a study on the ultrasound treatment of soybean isolates.

#### 3.1.7. Secondary Structure

The effect of ultrasonic treatment on the secondary structure of the protein was investigated by CD spectroscopy of SP. Figure 2C highlights two distinct minima near 208 nm and 222 nm, indicative of a predominance of α-helix structures in SP [32]. Figure 2D displays the composition of SP’s secondary structure, including α-helices, β-sheets, β-turns, and random coils. It was observed that following ultrasonication ranging from 0 to 500 W, there was a decrease in α-helix content from 47.4% to 40.9%. Concurrently, there was an increase in β-sheet and β-turn structures. This transformation can be attributed to the cavitation effect of ultrasonication, which alters the protein’s internal structure. The disruption of hydrogen bonds that stabilize the α-helix structures likely induces a shift towards β-turn and β-sheet structures, leading to the gradual unfolding of SP’s secondary structure from a tight helix [36,37].

### 3.2. Characterization of HIPEs

**Figure 3:**
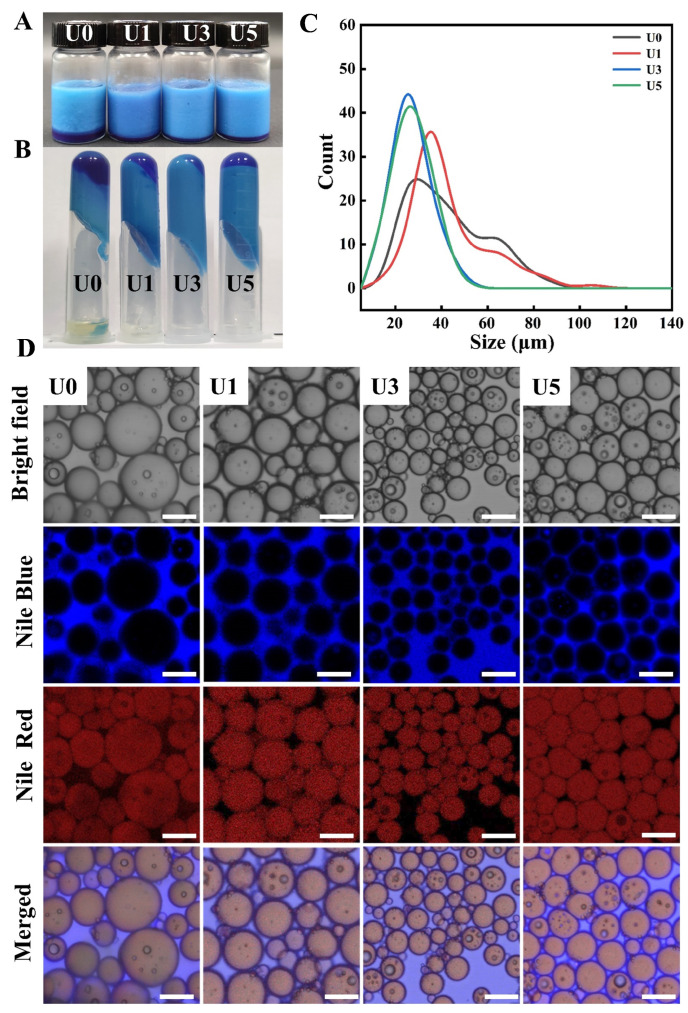
Appearance (**A**); Appearance after centrifugation (**B**); Particle size distribution (**C**); CLSM (**D**) of HIPEs stabilized by SP with different ultrasound treatments. Scale bar: 50 μm.

SP, subjected to various ultrasonic power treatments, was utilized to form the aqueous phase in the preparation of HIPEs. The appearance of these freshly prepared HIPEs after 2 h of resting is depicted in Figure 3A. HIPEs stabilized with U0 exhibited significant delamination after a 2 h rest, with the aqueous phase precipitating out. In contrast, emulsions stabilized with ultrasonically treated SP showed reduced water phase precipitation and increased emulsion layers. To accelerate the delamination process, the emulsions were centrifuged to assess their centrifugal stability. As shown in Figure 3B, after centrifugation, all emulsions showed delamination, from bottom to top, with a precipitated water phase, an intact emulsion phase, and an oil phase. Notably, the U0 stabilized emulsions showed the most pronounced delamination with more oil precipitated. In contrast, the amount of oil precipitated by the ultrasonic group was reduced and there was no oil leakage from the emulsions stabilized by U3 and U5. This suggests that the emulsions prepared by the ultrasonically treated SP have strong centrifugal stability. The microstructure of SP-stabilized HIPEs under different ultrasonic power treatments was further examined using CLSM, as shown in Figure 3D. The oil phase was dyed using Nile Red (resulting in a red fluorescence), whereas proteins were marked with Nile Blue (resulting in a blue fluorescence). The observation of blue fluorescence surrounding the red fluorescence established the adsorption of proteins onto the spherical droplets’ surface, providing evidence for the emulsion’s oil-in-water nature [38]. Additionally, CLSM images revealed a progressive decrease in droplet size and more homogeneous distribution with increasing ultrasonic power applied to SP. Statistical analysis of the emulsion droplet sizes is presented in Figure 3C. This analysis indicated that with increasing ultrasonic power, the particle size distribution gradually narrows and shifts to a higher, more singular peak, and the peaks at 20–40 µm shifted to the left, which indicated that the particle size was decreasing. This observation aligns with the findings from the microscopic images.

### 3.3. pH Stability of HIPEs

**Figure 4:**
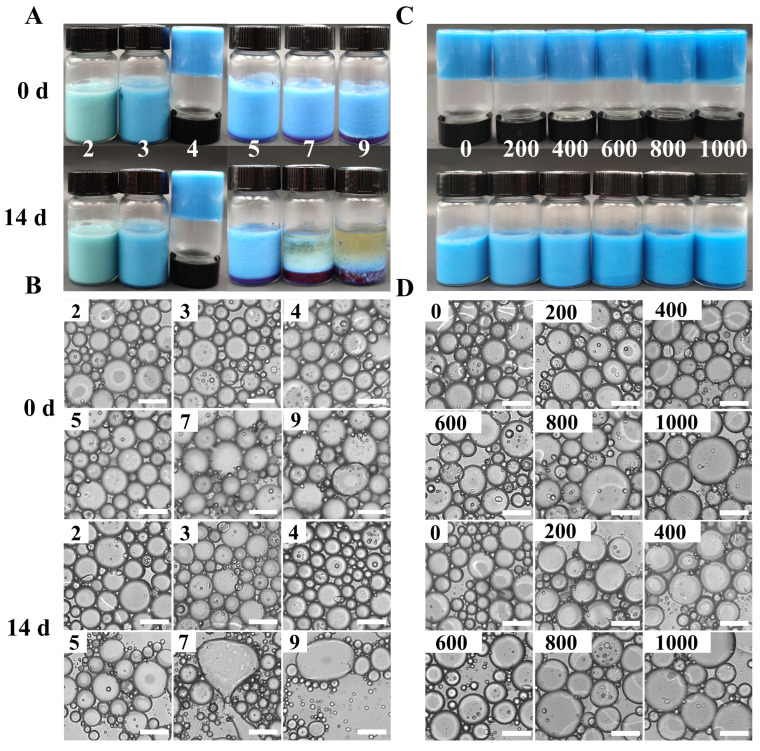
The effects of pH on the visual observation (**A**); Optical microscopy images (**B**); The effects of ionic strength on the visual observation (**C**); Optical microscopy images (**D**). Scale bar: 40 μm.

Emulsions stabilized with U3 showed the strongest stability and were therefore selected for further study. The pH value of emulsions is a key factor affecting their macroscopic properties and microstructure. Therefore, we observed the changes in the appearance and microstructure of emulsions before and after storage at different pH values. These changes are shown in Figure 4A. Visually, the emulsions under acidic conditions (pH 2 to 5) were relatively light in color and maintained their integrity after storage, and only a small amount of aqueous-phase precipitation was observed in the pH 5 emulsions. In contrast, emulsions with pH 7 and 9 showed emulsion breakage after 14 days of storage, when the oil and water phases separated. It is worth noting that the emulsion system did not flow when the sample bottle containing HIPEs at pH 4 was inverted, presumably forming a gel-like network structure within the emulsion. The microscopic morphology of the emulsions is shown in Figure 4B. In freshly prepared emulsions, the droplet size decreases with increasing pH in the low-pH range (2 to 4). The droplet size of emulsions with elevated pH (5 to 9) increased significantly. On the other hand, lower-pH emulsions remained stable in size and morphology even after 14 days of storage. However, at pH 7 and 9, the emulsion morphology becomes more dispersed with fewer droplets. This phenomenon may be because the change in pH environment affects the structure and emulsifying ability of SP, which leads to changes in the color and droplet size of the emulsions [28].

### 3.4. Ionic Strength Stability of HIPEs

Considering that salt is a common ingredient in food systems, we investigated the stability of the emulsions at different ionic strengths. Figure 4C shows the change in appearance of the emulsions at different ionic strengths. After 14 days of storage, slight delamination was observed in the emulsions with high ionic strengths (800 and 1000 mM), while no significant changes were found in the other groups. Microscopic observations of the emulsion droplets are shown in Figure 4D. It was found that the droplet size increased with increasing ionic strength. It is noteworthy that the emulsion droplets aggregated into larger droplets at higher ionic strengths. After 14 days of storage, the droplets became larger. This phenomenon can be attributed to the electrostatic shielding effect present in high ionic strength emulsions. This effect reduces the electrostatic repulsion between emulsion droplets [39], leading to an increase in the droplet polymerization size, and thus a decrease in emulsion stability.

### 3.5. Rheological Properties

#### 3.5.1. Rheological Properties of SP-Stabilized HIPEs with Different Ultrasound Treatments

**Figure 5:**
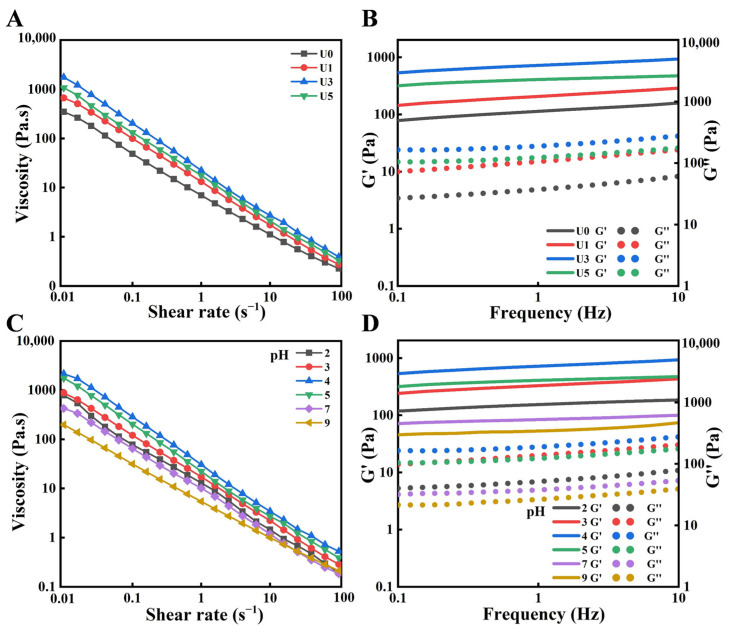
The rheological properties of SP-stabilized HIPEs with different ultrasonic powers: The changes in viscosity with the shear rate sweeps (**A**); The changes in storage (G′) and loss (G″) moduli with the angular frequency sweeps (**B**); The rheological properties of U3-stabilized HIPEs at different pH conditions: The changes in viscosity with the shear rate sweeps (**C**); The changes in storage (G′) and loss (G″) moduli with the angular frequency sweeps (**D**).

In addition to macroscopic changes and microstructural changes, rheological properties are important indicators for evaluating the performance of emulsions. In this study, the rheological properties of HIPEs were investigated by measuring viscosity, storage modulus (G′), and loss modulus (G″). Figure 5 shows that the apparent viscosity of all the emulsion samples decreases with increasing shear rate, exhibiting the shear-thinning behavior characteristic of non-Newtonian fluids. This behavior may be due to structural damage and deformation of droplets at high shear rates [28]. The apparent viscosity of the ultrasonically treated SP-stabilized emulsions was higher than that of the untreated group, with the U3-stabilized emulsions having the highest viscosity. This result contrasts with the trend in emulsion droplet size. In a study of ultrasound-treated quinoa protein-stabilized emulsions, it was found that ultrasound treatment led to the formation of stronger flocculated structures around the droplets, which effectively resisted structural disruption at high shear rates [40]. In addition, the decrease in droplet size increases the number of friction points and enhances the interaction force between the droplets, thus increasing the viscosity of the emulsion [41]. Figure 5B depicts the stock G′ and G″ of SP stabilized emulsions treated with different ultrasonic powers. Throughout the frequency range, G′ is consistently greater than G″, indicating that the emulsion has the properties of an elastic gel. This is the main characteristic of protein-stabilized HIPEs [27]. The higher the G′ value, the greater the gel strength and the greater the resistance of the emulsion to external stresses, leading to greater stability [42]. The above results indicate that ultrasonic treatment can give higher gel strength and improve the viscoelasticity of the emulsion.

#### 3.5.2. Rheological Properties of HIPEs at Different pHs

The change in viscosity of U3-stabilized emulsions at different pH values is shown in Figure 5C, where all samples exhibit shear-thinning behavior with no change in emulsion type. The apparent viscosity under acidic conditions is higher than that under alkaline conditions, and this result is related to the droplet morphology of the emulsion. Figure 5D shows the G′ and G″ of emulsions at different pH conditions. As shown, the emulsion with pH 4 has the highest G′ and G″ values. Both G′ and G″ values decrease with increasing pH above 4, indicating a weakening of the emulsion structure, which is in agreement with the observations made by optical microscopy. The rheological properties of HIPEs are related to droplet size and interactions; smaller, more numerous droplets form a tighter structure, which increases resistance to external forces [43]. In conclusion, rheological characterization further validated the changes observed in the appearance and microstructure of HIPEs. In particular, HIPEs exhibited excellent gel properties and stability at pH 4 and were therefore selected for subsequent experiments.

### 3.6. Protective Effect of Emulsion on Curcumin

**Figure 6:**
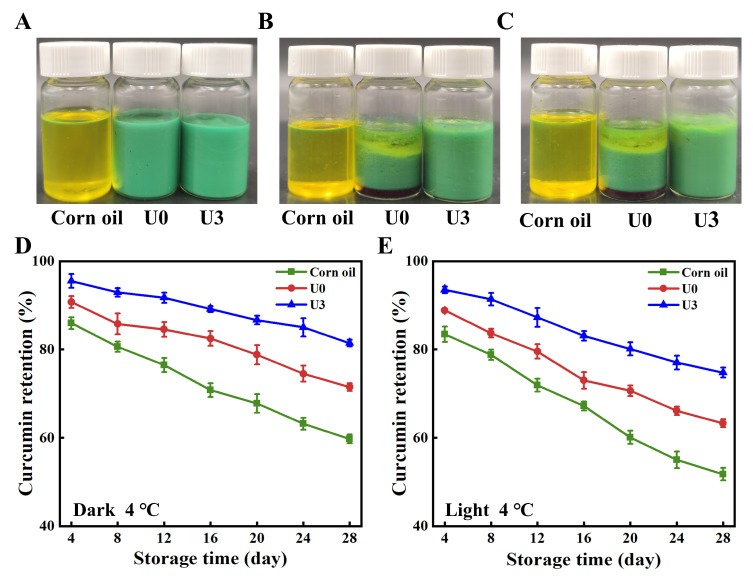
Changes in the appearance of curcumin stored in corn oil and U0- and U3-stabilized HIPE under different environmental conditions for 28 days: Fresh Samples (**A**); Samples after 28 days of dark storage (**B**); Samples after 28 days of light storage (**C**); Retention of curcumin in dark environments (**D**); Retention of curcumin in light environments (**E**).

To assess the protective effect of emulsions on curcumin, we incorporated curcumin into corn oil and prepared HIPEs loaded with curcumin. We then examined the changes in appearance of both curcumin-loaded corn oil and the curcumin-loaded emulsion after 28 days of storage, as shown in Figure 6A–C. Over this period, the color of the curcumin-loaded emulsion shifted from green to yellowish-green, and the U0-stabilized emulsion exhibited signs of breakage. Additionally, we measured the retention of curcumin in these emulsions over 28 days under both dark and ambient light conditions. In dark conditions at 4 °C, the retention of curcumin in the U3-stabilized emulsion was 81.43 ± 0.75%, which was higher compared to 71.49 ± 0.88% in the U0-stabilized emulsion and 59.75 ± 1.01% in curcumin-infused corn oil alone (Figure 6D). Under light exposure at 4 °C, as depicted in Figure 6E, curcumin in corn oil alone retained only 51.76 ± 1.42%, whereas curcumin encapsulated in HIPEs exhibited better protection. In conclusion, the retention of curcumin was more effectively preserved in U3-stabilized HIPEs compared to U0-stabilized emulsions and corn oil alone across different storage conditions. HIPEs loaded with curcumin demonstrated superior retention, particularly in dark environments.

### 3.7. In Vitro Simulated Digestion

**Figure 7:**
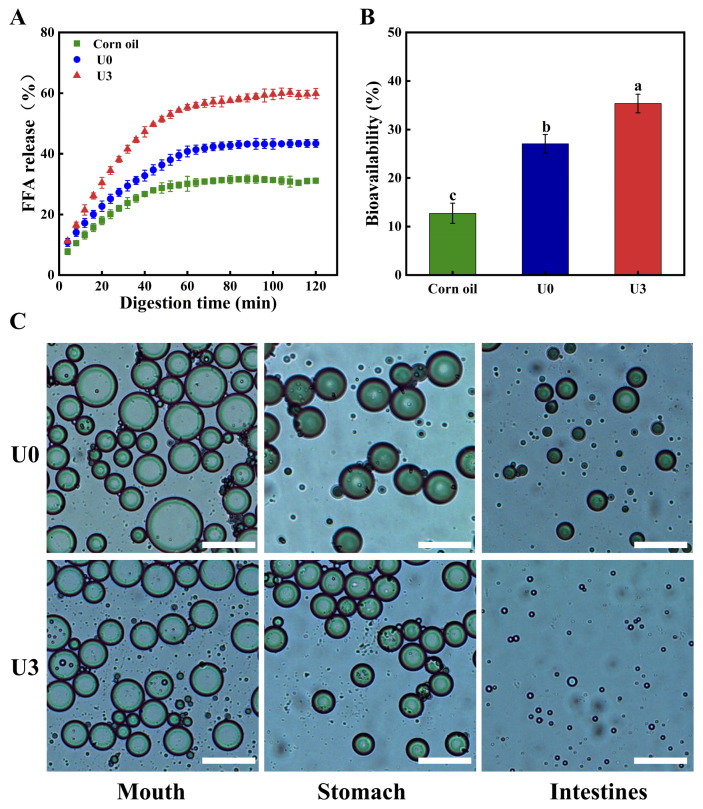
Rates of FFA release from corn oil and HIPEs during intestinal digestion (**A**); Bioavailability of curcumin in corn oil and HIPEs (**B**); Microstructure of curcumin-loaded HIPEs stabilized by U0 and U3 at different stages during in vitro digestion (**C**); Scale bar: 100 μm. Different lowercase letters in the column diagram suggest significant differences (*p* < 0.05).

The Free Fatty Acid (FFA) release rates of the samples were determined by mixing SP-stabilized HIPEs and corn oil with a simulated digestion solution. During the initial 40 min, rapid digestion of lipids occurred across all samples, corresponding with the swift adsorption of lipase at the oil–water interface. This process reached a peak at 60 min, after which the digestion rate began to decline, eventually stabilizing. The fatty acid release rates post-digestion were in the following order: U3-stabilized emulsions (59.84 ± 1.71%) > U0-stabilized emulsions (43.39 ± 1.31%) > corn oil (31.09 ± 0.49%), with U3-stabilized HIPEs exhibiting the fastest release rate. The structural characteristics and droplet size of the emulsion are key factors influencing FFA release [44]. Figure 7C displays the changes in droplet morphology during digestion. For post-saliva digestion, the morphology of both U0- and U3-stabilized emulsions remained unchanged. Subsequent digestion by gastric juices resulted in the formation of relatively small droplets. Following intestinal digestion, the droplets of the U3-stabilized emulsion were significantly smaller than those in the U0-stabilized emulsion. Smaller droplets have more surface area, which facilitates their interaction with bile salts and lipases present in the digestive fluid. This interaction ultimately leads to an increased rate of FFA release [45]. Additionally, the bioavailability of curcumin in HIPEs and corn oil was evaluated by measuring the curcumin content in the intermediate micellar layer obtained after centrifuging the digestate. As shown in Figure 7B, the bioavailability of curcumin in the U3-stabilized emulsion (35.36 ± 1.92%) was greater than that in the U0-stabilized emulsion (27.04 ± 1.91%) and corn oil alone (12.73 ± 2.08%). This trend aligns with the FFA release rates observed. The higher bioavailability in U3-stabilized emulsions could be attributed to their smaller droplet size, facilitating a quicker FFA release and more efficient conversion of the curcumin-loaded oil phase into micelles, resulting in enhanced curcumin bioavailability [46]. A similar increase in bioavailability of β-carotene was observed in emulsion gels prepared from β-accompanied soybean globulin after ultrasound and pH treatment, where the reduction in droplet size was a contributing factor [47]. These findings suggest that ultrasound-treated protein-stabilized HIPEs can effectively deliver bioactive compounds, holding promising potential for food industry applications.

## 4. Conclusions

This study demonstrates that ultrasonic treatment significantly altered the original morphology, reduced particle size, and enhanced the surface hydrophobicity of SP, achieving optimal results at 300 W with a three-phase contact angle of 84.3°. Next, HIPEs were prepared using SP, and it was found that ultrasonic treatment significantly enhanced the emulsification ability of SP with smaller and more uniformly distributed droplets in its stabilized emulsion. The emulsions not only had higher apparent viscosity and modulus values (G′ and G″) but also enhanced centrifugal stability. In addition, U3-stabilized HIPEs have excellent storage stability and significantly improve curcumin retention and bioavailability. These findings suggest that ultrasound-modified SP is an exceptional emulsifier, and its stabilized HIPEs have the potential to more effectively encapsulate and deliver hydrophobic nutrients such as curcumin.

## Data Availability

The data presented in this study are available on request from the corresponding author. The data are not publicly available due to the need for the first author to apply for their master’s degree.
